# Background matching through fast and reversible melanin-based pigmentation plasticity in tadpoles comes with morphological and antioxidant changes

**DOI:** 10.1038/s41598-023-39107-4

**Published:** 2023-07-26

**Authors:** H. Christoph Liedtke, Karem Lopez-Hervas, Ismael Galván, Nuria Polo-Cavia, Ivan Gomez-Mestre

**Affiliations:** 1 Ecology Evolution and Development Group. Biological Station of Doñana – CSIC, 41092 Seville, Spain; 2 Max Planck Institute for Evolutionary Biology, August-Thienemann Str. 2, 24306 Plön, Germany; 3 Department of Evolutionary Ecology, National Museum of Natural Sciences, CSIC, 28006 Madrid, Spain; 4 Department of Biology, Universidad Autónoma de Madrid, Ciudad Universitaria de Cantoblanco, 28049 Madrid, Spain

**Keywords:** Animal physiology, Ecology, Evolution, Ecology

## Abstract

Facultative colour change is widespread in the animal kingdom, and has been documented in many distantly related amphibians. However, experimental data testing the extent of facultative colour change, and associated physiological and morphological implications are comparatively scarce. Background matching in the face of spatial and temporal environmental variation is thought to be an important proximate function of colour change in aquatic amphibian larvae. This is particularly relevant for species with long larval periods such as the western spadefoot toad, *Pelobates cultripes*, whose tadpoles spend up to six months developing in temporary waterbodies with temporally variable vegetation. By rearing tadpoles on different coloured backgrounds, we show that *P. cultripes* larvae can regulate pigmentation to track fine-grained differences in background brightness, but not hue or saturation. We found that colour change is rapid, reversible, and primarily achieved through changes in the quantity of eumelanin in the skin. We show that this increased eumelanin production and/or maintenance is also correlated with changes in morphology and oxidative stress, with more pigmented tadpoles growing larger tail fins and having an improved redox status.

## Introduction

Crypsis through background matching is widespread in the animal kingdom^1,2^. For crypsis to be effective, selection must act on phenotypes to minimize the signal-to-noise ratio between the organism and its background^3^. This can be challenging, given that backgrounds need not be static. A species habitat can be temporally and spatially heterogeneous. Background choice behaviour^4,5^, geographic polymorphism^6^ or colour change^1,7–9^ are examples of adaptive strategies that may have evolved to improve concealment in such heterogeneous circumstances.

Colour change is a remarkable form of phenotypic plasticity and can occur at varying rates, from changes in seasonal phenotypes to near instantaneous shifts^10,11^. Its function for improving crypsis has been documented in a broad range of taxa^2^, spanning multiple classes of both aquatic and terrestrial vertebrates and invertebrates, from reptiles^12^, mammals and birds^13^ to cephalopods^14^ and arthropods^15^. However, its underlying mechanisms and the selective pressures driving the evolution of pigmentation plasticity are complex and not yet well understood^10^. Although slow colour change seems to be more common^11^, our knowledge on the sensitivity of pigmentation plasticity and its associated physiological costs are largely restricted to a few organisms, typically those that can change colour rapidly e.g.^16^. Experimental data testing phenotypic responses to different albedos across a broader taxonomic range is therefore needed to further our understanding of how such a complex trait operates.

Colour change has frequently been reported in amphibians, both in adults and larvae^13–22^. In adults, colour change can be elicited by environmental stimuli such as background colour, light intensity, temperature and humidity^7,17–22^, along with stress^23^, intra-specific signalling^24,25^ and ontogenetic changes^26,27^. In larvae, background matching to evade visual predators may be the most important role^28,29^. This is because larvae tend to be more diurnal than adults^30^, and sexual selection (often involved in the evolution of pigmentation) is largely irrelevant^31^.

Plasticity of any kind however, is expected to incur physiological costs^10,11,32^ and so the hypothesized advantages of colour change may need to be balanced with associated trade-offs. Relatively little is known about the direct energetic costs of the synthesis and/or rearrangement of pigments in pigment cells (chromatophores)^2,33^, but studies have highlighted important physiological consequences. In birds for example, the synthesis of pheomelanin, one of the two chemical forms of melanin, requires the consumption of the amino acid cysteine and its physiological reservoir, the intracellular antioxidant glutathione^33^. More directly, newt larvae have shown increased metabolic rates associated with increased pigmentation^28^. If enhanced metabolic rate is sustained, the production of reactive oxygen species (ROS) will be increased, and so will the need to minimise their harmful oxidative effects on tissues^34,35^. Moreover, chromatophores and pigment production are controlled by a multitude of hormones, some of which are involved in other developmental pathways^36,37^. For example, elevated levels of corticosterone, the amphibian stress hormone, has been associated with light backgrounds^38^ and interference with melanin synthesis^39^. Corticosterone is also an important morphogen known to have wide-ranging effects on behaviour, development and morphology of amphibian larvae^40,41^. As a result, changes in melanin-based pigmentation in tadpoles may be accompanied by changes in other aspects of development and morphology e.g.^39^.

Here, we perform a series of experiments to test the extent, speed and sensitivity of colour change in aquatic larvae of the Western Spadefoot toad (*Pelobates cultripes*), and associated physiological and morphological implications. By exposing tadpoles to different grey-scale backgrounds (different brightness) we test whether pigmentation is a discrete threshold trait (i.e. polyphenic) or is continuously regulated. We also expose tadpoles to backgrounds of three different colours; red, green and blue, to test whether colour change is limited to changes in saturation and brightness or whether it also extends to changes in hue. By reciprocally transplanting tadpoles reared on dark and light backgrounds, we test whether changes in pigmentation are ontogenetically fixed or whether they are reversible, and at what rate colour change occurs. We investigate the association between increased pigmentation and morphology (body shape), and test for indirect costs of colour change by quantifying changes in the production of oxidative damage (malondialdehyde) and antioxidant capacity. Finally, we use Raman spectroscopy to identify the dominant pigment type underlying this facultative colour change.

## Results

### Dorsal pigmentation plasticity

**Figure 1:**
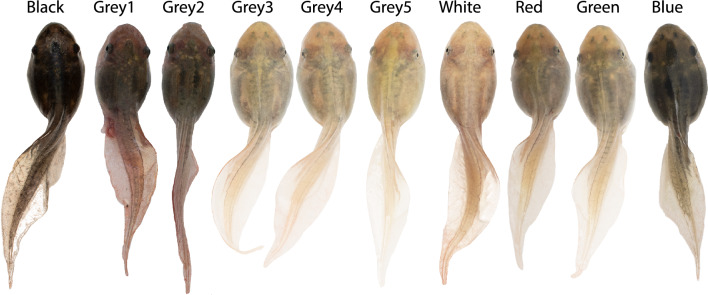
Images of *Pelobates cultripes* tadpoles exemplifying changes in the degree of pigmentation induced by rearing on different backgrounds. Tadpoles were reared on black, white, red and blue backgrounds and five shades of grey, becoming increasingly lighter from grey 1 to grey 5.

**Figure 2:**
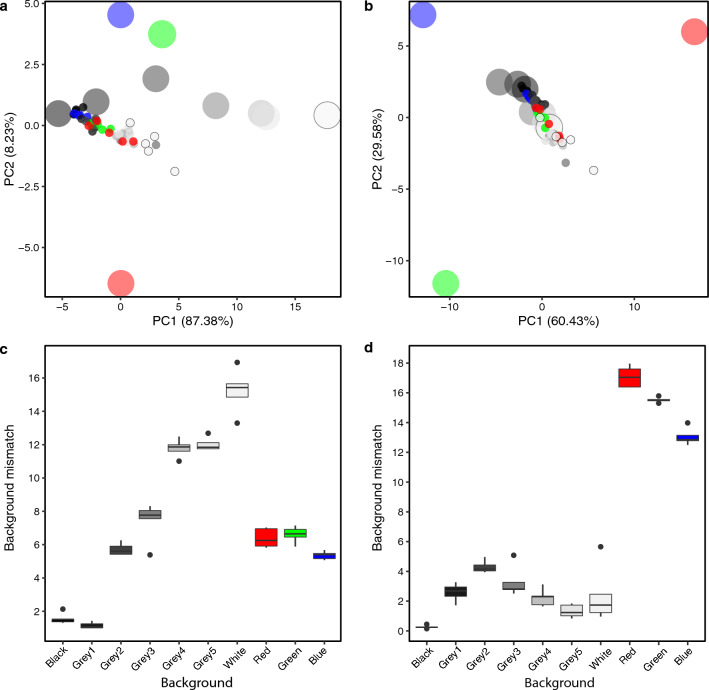
First two axes of principal component analyses for non-normalized (**a**) and normalized (**b**) photospectral compositions. The plots show the positions in the colour space of the tadpole dorsal pigmentation (small points) relative to the position of the background (large points of the same colour) that tadpoles were reared on. Fill colours represent the background colours that tadpoles were reared on. For example, large red point represents the mean colour measurement of the red containers, and the five small red points represent the colour measurements of the tadpoles reared in this red container. The boxplots (**c**,**d**) show Euclidean distances in colour space between the tadpole pigmentation and their corresponding background (distance between smaller and larger points in the PCA). Larger distances therefore represent greater mismatch between the tadpole dorsal colour and their corresponding background.

Rearing *Pelobates cultripes* tadpoles on different backgrounds of differing brightness induced changes in the degrees of pigmentation of the dorsum (Fig. 1). We used a principal component analyses (PCA) to compare the spectral composition of tadpoles and their corresponding backgrounds, measured with a handheld photospectrometer. The first component of the PCA on untransformed spectra (explaining 87.4% of the variance; Fig. 2a) is evenly loaded by reflectance measurements at all wavelengths and therefore represents differences in the overall area under the reflectance curve, i.e. brightness. The second principal component (explaining 8.2% of the variance) differentiates high from low wavelength reflectance. Accordingly, PC1 separates the grey-scale backgrounds (the large black, grey and white points in Fig. 2a) and PC2 separates the blue and green (shorter wavelengths) from the red (longer wavelengths) backgrounds. The tadpoles reared on these different backgrounds (smaller points of corresponding colours on Fig. 2a) are primarily separated along PC1. Normalizing spectra to standardize overall brightness results in the maximal separation of peak wavelengths (the red, green and blue backgrounds), with all grey-scale backgrounds clustered together (Fig. 2b). Here too, however, tadpoles separate out only along the grey-scale axis.

The distance between the background and the tadpoles in the PCA space was used to quantify the degree of background-mismatching (Fig. 2c,d). For the unnormalized spectra, where the dominant factor is brightness, the least mismatch was recovered for tadpoles reared on darker backgrounds (Fig. 2c). The degree of mismatch increased with increasing background brightness (Fig. 2c). In contrast, for the normalized spectra, the greatest mismatch is recovered for tadpoles reared on the red, green and blue backgrounds, with mismatch for grey-scale backgrounds being comparable across all shades of grey (Fig. 2d).

**Figure 3:**
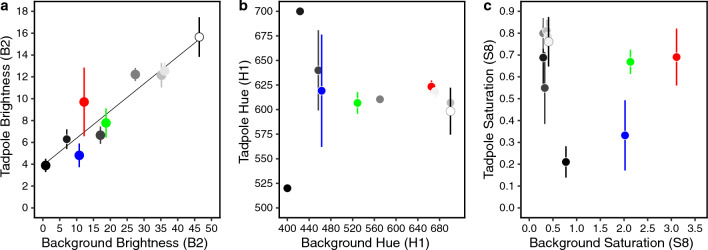
Colour summary statistics for (**a**) brightness (B2), (**b**) hue (H1) and (**c**) saturation (S8). B2 is the mean relative reflectance over the entire spectral range (%), H1 is the maximum peak of reflectance in the wavelength (nm) and S8 is the difference between the maximum and minimum reflectance divided by the mean reflectance. These indices for tadpole dorsal skin (y axis) are contrasted against the same indices of the container in which they were reared (x axis). Points represent means, and error bars show standard deviation of five measurements per treatment. Fill colours represent the background colours that tadpoles were reared on (red, green, blue, black, white and five shades of grey). The significant regression line is shown for B2 (p < 0.001). H1 and S8 showed no significant correlations.

Summarizing the spectra as statistics representative of hue (H1), saturation (S8) and brightness (B2) confirm the above observations. Tadpole brightness (B2) is strongly, positively correlated with background brightness (adjusted R^2^ = 0.846, F = 50.58, Df = 1,8, p < 0.001; Fig. 3a), but hue (H1) and saturation (S8) are not (adjusted R^2^ = − 0.125, F = 0.00009, Df = 1,8, p = 0.993 and adjusted R^2^ = − 0.079, F = 0.343, Df = 1,8, p = 0.574 respectively; Fig. 3b,c). Hue and saturation are calculated based on maximum and minimum reflectance values. Spectra of the darkest tadpoles (reared on black and darkest grey backgrounds) are flat, with no clear peak reflectance (Supplementary Fig. 1). This makes their H1 and S8 measurements more sensitive to extreme values. Removing these, however, does not affect the results (hue: adjusted R^2^ = − 0.098, F = 0.377, Df = 1,6, p = 0.562; saturation: adjusted R^2^ = 0.005, F = 1.03, Df = 1,6, p = 0.349).

### Pigmentation reversibility

**Figure 4:**
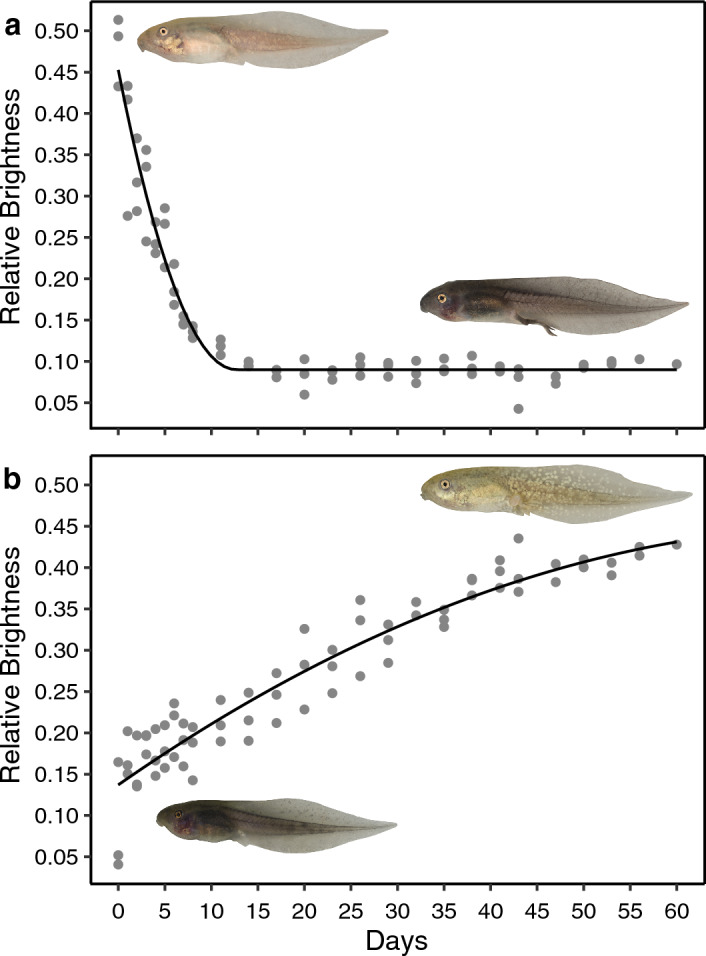
Relative brightness changes (in luma) of tadpole dorsal pigmentation over time, for (**a**) light tadpoles transplanted onto black backgrounds and (**b**) dark tadpoles transplanted onto white backgrounds. Points show tadpole measurements (n = 3 per transplant) and lines show nonlinear regression slopes. Inserted photographs show exemplary tadpoles at day 0 and then the same tadpole after 60 days.

Tadpoles in late stages of development (Gosner stage 35 onwards) were able to reverse pigmentation induced by previous rearing conditions (Fig. 4). Light tadpoles reared in white containers which were transplanted to black containers were able to completely darken their dorsal pigmentation (Fig. 4a; Supplementary Video 1), and vice versa (Fig. 4b; Supplementary Video 2). A quadratic plateau model for the light-to-dark transition (Fig. 4a) estimated the intersect at 0.451 luma (i.e. the lightest point) with a slope estimate of -0.057 and a plateau breakpoint at 12.673 days and 0.087 luma (the darkest point). For the dark-to-light transition (Fig. 4b), the intersect was estimated at 0.137 luma with a slope estimate of 0.007 and a plateau breakpoint at 79.741 days and 0.451 luma. The rate of darkening was therefore noticeably faster than the rate of lightening, with the maximum lightening of dark tadpoles predicted to occur after the experiment was terminated. The estimated plateau points (0.087 luma for the darkened tadpoles and 0.451 for the lightened tadpoles) are highly comparable to the estimated start points of the lightening and darkening tadpoles (intersects; 0.137 and 0.451 luma respectively).

### Morphology

**Figure 5:**
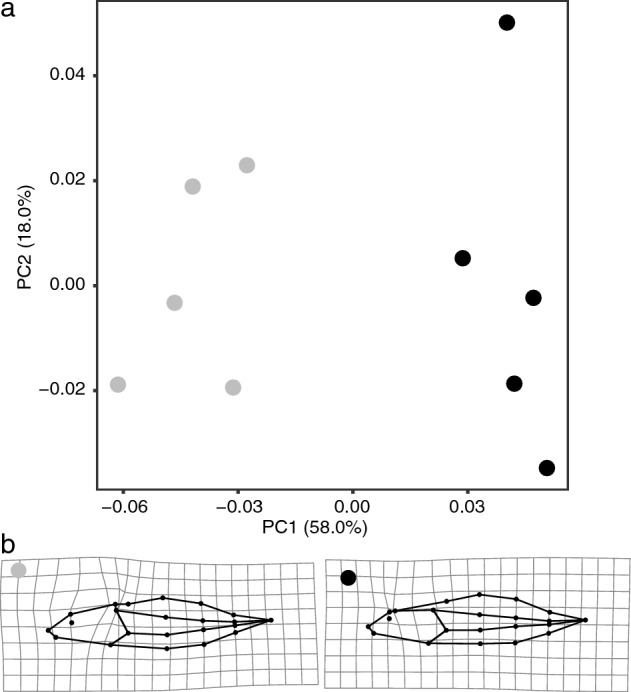
Comparison of body shapes of dark versus light tadpoles, where (**a**) shows the first two axes of a Principal Component Analysis (PCA) on Procrustes shape coordinates, and (**b**) shows the average landmark positions with wire-frames of light (left) and dark (right) tadpole morphologies. Point colour distinguishes light (grey) from dark (black) tadpoles.

Dark and light tadpoles significantly differed in body shape. The first principal component (explaining 58.0% of the overall variance) of a PCA representing morphological shape space, separates the dark from the light tadpoles (Fig. 5a). The Procrustes ANOVA supported a significant treatment effect of tadpole colour on tadpole shape (F = 9.804, Z = 2.487, Df = 1,8, p = 0.007). The first principal component in shape variation mainly represented changes in the position and angle of insertion of the tail fin on the dorsum of tadpoles. Dark tadpoles therefore present a more anterior insertion and a slightly deeper tail fin than light tadpoles (Fig. 5b).

### Oxidative stress

**Figure 6:**
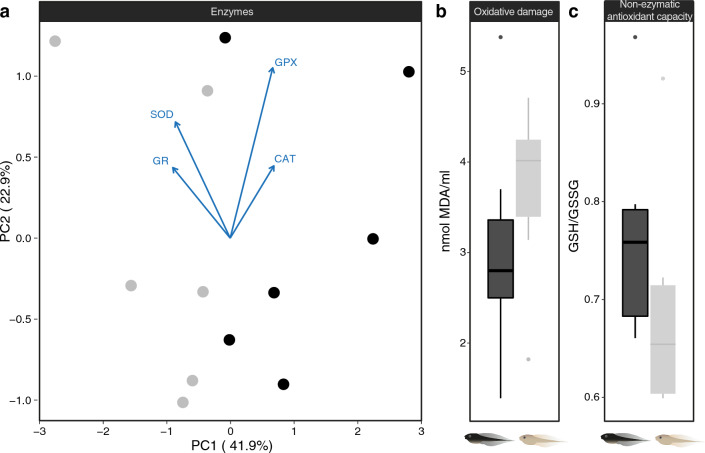
Indicators of oxidative stress. (**a**) First two PCA axes of oxidative stress enzyme concentrations. Arrows show loadings of the four enzymes: superoxide dismutase (SOD), catalase (CAT), glutathione reductase (GR) and glutathione peroxidase (GPx). Also shown are boxplots of (**b**) malondialdehyde concentration (nmol MDA/ml) and (**c**) of ratio of reduced glutatione (GSH) to oxidazed glutathione (GSSG). Black versus grey symbols and illustrations refer to dark versus light tadpoles, where n = 5 per group.

Antioxidant enzyme activity was markedly distinct between dark and light tadpoles. A PCA on enzyme activity separated out tadpoles by treatment along the first component axis, which explained 41.9% of the variance (Fig. 6a). Darker tadpoles had higher glutathione peroxidase and catalase activity, but lower superoxide dismutase and glutathione reductase activity compared to light tadpoles (Fig. 6a; PC1 *t* value = 3.491, p-value = 0.006). Dark tadpoles showed a trend towards lower oxidative damage (MAD; *t* value = − 0.512, p-value = 0.62) and higher non-enzymatic antioxidant capacity (GSH/GSSG ratio; *t* value = 1.09, p-value = 0.301) compared to light tadpoles (Fig. 6b,c). However, these differences were not significant.

### Raman spectroscopy

**Figure 7:**
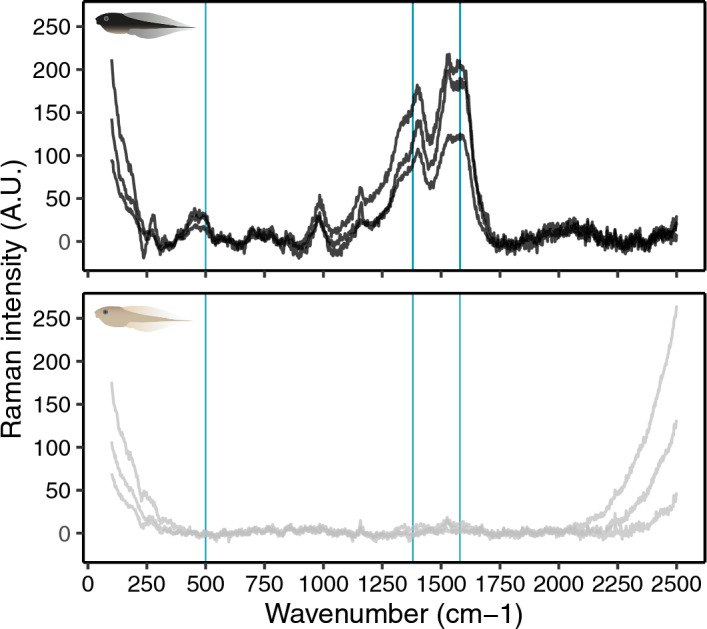
Raman spectra of dorsal skin of dark (top panel) and light (bottom panel) tadpoles. Raw spectra are shown for each of three dark and three light tadpoles. Blue vertical lines indicate diagnostic band positions characteristic of eumelanin (peaks at 500, 1380 and 1580 cm^–1^) that are present in the dark, but not the light tadpoles.

We detected Raman signals characteristic of eumelanin in the dorsal skin of all dark tadpoles with these being absent in the light tadpoles (Fig. 7). The Raman spectra showed strong peaks close to 1380 and 1580 cm^–1^ and a weaker peak at 500 cm^–1^. This profile resembles the D and G bands of disordered graphite, characteristic of eumelanin^42^. We did not detect signals for other forms of melanin, such as pheomelanin, which has Raman bands at approximately 500, 1490 and 2000 cm^–1^^42^.

## Discussion

Natural observations of tadpoles with varying degrees of pigmentation are widely documented^29,43^, but experimental data studying facultative colour change in amphibian larvae are comparatively scarce. Here, we find that tadpoles of *Pelobates cultripes* significantly alter their pigmentation in response to background brightness in a matter of days, but not hue or saturation. Moreover, pigmentation adjustments are continuous, tracking everything from slight changes in background brightness to complete inversions in brightness. That being said, tadpoles can better match dark compared to light backgrounds and darkening is faster than lightening. We demonstrate that this colour change is mostly driven by changes in the extent of melanisation, specifically of eumelanin, and increased pigmentation is accompanied by changes in morphology (deeper, more anteriorly inserted tail fins) and an improved redox status.

We show that tadpoles were significantly more pigmented in darker environments. This same pigmentation change has been observed in a number of distantly related amphibian larvae^7,17,32,40,45,46^ and may therefore be a conserved, effective strategy for increased concealment from visual predators^28,44^. However, it is not universal and seems to be stage-dependent, with some species capable of altering pigmentation more, or at earlier stages than others^45^. Interestingly however, we go on to show that background matching is limited in at least three ways in this species. Firstly, concealment is best in darker environments and background-tadpole colour mismatching increases the brighter the background. Ineffective crypsis on light backgrounds has been noted in other species too^46^, and a common problem for crypsis on light backgrounds may be that certain vital molecules such as haemoglobin are conspicuously coloured. Some amphibians have nonetheless evolved fascinating ways to circumvent such limitations, by having white or reflective peritonea covering dark organs, or by actively reducing the number of red blood cells circulating in the body^47^.

Secondly, darkening is faster than lightening. This reversibility of darkening and lightening, also observed in other amphibians^28,46,48^, is relatively fast, with measurable changes in pigmentation occurring within the first 24 h and maximum pigmentation change reached in under two weeks. This rate of colour change may be particularly ecologically meaningful for temporal heterogeneity of the environment. *Pelobates cultripes* are among the first anurans to lay eggs in the season, shortly after the formation of temporary ponds and have a long larval period that can easily span six months. The life-cycle of these ponds over a period of several months involves seasonal light differences, changing vegetation density and type, water turbidity and detritus and depth. The rates of darkening and lightening are asymmetric however, with lightening taking up to six times as long as darkening. To test the robustness of this finding, and whether this is indicative of different processes involved needs further investigation, and a more statistically meaningful sample size. Similarly, pigmentation in tadpoles may also change ontogenetically^46^. Although we have not observed this to be the case in *P. cultripes* to a degree comparable to background-induced colour change, future studies should include control groups to distinguish between such ontogenetic and background-driven colour change.

Qualitative observations suggest that darkening was largely achieved through a gradual, dorsal-to-ventral extension of dark pigments, lightening was achieved through the gradual, uniform loss of dark pigments as well as topical growths of reflective patches of lighter pigments. Pigment synthesis and degradation may therefore be similar to long-term background adaptation in teleost fish, in which chromatophores originate in the neural crest and differentiate during their migration to various tissues and regions. In reverse, chromatophore degradation is achieved through apoptosis and a more even discharge of melanin from the skin surface^49^.

Thirdly, colour change is limited to a single colour axis. Pigmentation in amphibians is controlled by different type of chromatophores: melanophores, xanthophores and iridophores^50,51^, which produce different pigments. The photospectrometry analyses of dorsal colouration however suggested that *P. cultripes* tadpoles are primarily able to achieve colour change through changes in brightness, but not in hue or saturation. Using Raman spectroscopy, we were able to confirm that the darkening in *P. cultripes* skin is achieved primarily through increasing the quantity of eumelanin and the absence of other pigments. Noticeable was the lack of detection of pheomelanin across all samples, a pigment responsible for yellow–red colours in vertebrates. This is in line with the assumption that like fish, amphibians may not be able to produce this type of melanin^52,53^ (but see^54^). Our findings therefore conform to the idea that colour change in *P. cultripes* is achieved by principally only regulating the synthesis or arrangement of a single pigment type. This is nonetheless surprising, given that plastic changes in amphibian colouration are known from other contexts, such as shifts in hue during ontogeny^26^ and in response to predation risk^43,55–57^. This extends beyond amphibians, with groups across the tree of life showing the capacity for changes in hue (e.g. arthropods^58^, cephalopods^59^ and reptiles^60^).

Nonetheless, changes primarily in brightness over other colour attributes is not uncommon either. This has been documented in adult midwife toads^22^, rock goby fish^61^ and shore crabs^62^. This could suggest that brightness is an attribute of colour that may be physiologically or evolutionarily less costly to regulate than hue, or that matching of brightness is a more efficient way to achieve crypsis than matching hue or saturation. In accordance with this idea, modelling visual detection of crabs by birds for example, has shown that mismatches with background brightness is more easily perceived by this predator than mismatches in hue^62^.

We observed a significant change in body shape between dark and light tadpoles, with darker tadpoles showing a more anterior insertion of the tailfin. At the same time, we observed that increased pigmentation is correlated with a reduction in glutathione reductase activity and in the level of malondialdehyde, indicative of an improved redox status. Interestingly, this same phenotypic and physiological shift is induced in response to increased predation risk^63–65^. Perceived predation risk, often via water-borne chemical cues, is known to induce reduced activity levels and metabolic rate in amphibian larvae as well^66–68^. A recent study found that melanisation in *Microhyla fissipes* tadpoles^39^ was also associated with changes in morphology (shorter tails) and was interfered by addition of exogenous corticosterone^39^. Corticosterone level is positively associated with metabolism and is also downregulated in the presence of predators^64,69^. This is again consistent with the metabolic consequences of exposure to predator cues in spadefoot toad tadpoles. This hints towards a link (direct or indirect) between processes that regulate pigmentation and stress-induced changes in phenotypes and physiology^39^. However, whether oxidative stress is correlated specifically with increased pigmentation or more generally with alteration in pigmentation needs to be further investigated^32^. In the same way, the involvement of corticosterone in regulating melanisation may also impact overall developmental rate, and therefore size and weight at metamorphosis. Such carry-over effects may be interesting to study in the future.

In summary, an increasing number of studies are showing that pigmentation, body shape and oxidative stress may be physiologically linked^22,32,39,46^ and we hypothesize that the same, or components of the same, hormonal pathways may be involved in governing pigmentation and morphological plasticity. This may have important implications for adaptive hypotheses of deeper tails being a trait that has been selected exclusively for reducing the risk of predation.

## Methods

### Tadpole husbandry

We collected egg clutches of *Pelobates cultripes* from a temporary pond in Doñana National Park (Laguna del Zahíllo; GPS: 36.987492, − 6.506652, Huelva, Spain). The microhabitat consisted of a temporary pond with sandy soil, fully exposed without canopy cover, but with shrubs along the shore and patches of dense aquatic vegetation (dominated by *Myriophyllum alterniflorum* and *Ranunculus peltatus*) that result in a mosaic of dark and light microhabitats. The clutches were transported to Doñana Biological Station in Seville where they were kept in shallow, white plastic trays with dechlorinated tap water. A few days after tadpoles hatched and started feeding (Gosner Stage 25^70^), we haphazardly selected larvae to be used in the experiments and placed them in individual 2.5 L plastic containers of different colours as detailed below. Tadpoles were maintained at 20 °C with a 12 h:12 h photoperiod in a climatic chamber, with two fluorescent tubes fixed 30 cm above the water surface as the primary light source. Throughout the whole experiment, tadpoles were fed with ground-up rabbit chow ad libitum three times per week, and the water was replaced twice a week. The climate chambers should create homogenous environments across all treatments. Nonetheless, containers with tadpoles were arranged in a systematic design so that each experimental group had the same number of tadpoles exposed to the front and back of each chamber, to reduce the possibility of unmeasured, environmental variation introducing bias in the study. Due to the reduced sample sizes, this was deemed more appropriate than a random design. Sample sizes (stated in each section below) were kept to a minimum to reduce the number of animals needed, but maintain sufficient statistical power. In total, 74 tadpoles were reared in the experiment.

Permission for collecting egg clutches in Doñana National Park were issued by the Junta de Andalucía. The procedures for rearing and manipulation of tadpoles were approved by Dirección General de la Producción Agrícola y Ganadera, and the IACUC committee of Doñana Biological Station. All experiments were performed in accordance with the relevant guidelines and regulations set out by these committees. The procedures were conducted in accordance with the ARRIVE guidelines (https://arriveguidelines.org/arrive-guidelines).

### Dorsal pigmentation plasticity

We tested the sensitivity of tadpoles to a gradient of environmental albedo. To achieve this, we raised tadpoles in a set of grey-scale (black, five incremental shades of grey, and white) and three different colour (red, green and blue) backgrounds. To manipulate the environment in this way, tadpoles were reared in 2.5 L clear containers that were spray-painted with different colours on the outside. The brand and colour code of the spray paint used is provided in Supplementary Table 1. Each treatment (background) was replicated five times. As such, 50 tadpoles were reared in total, five per each of ten backgrounds.

The tadpoles were at Gosner stage 25 when starting the experiment and reared on their designated backgrounds for 40 days. At the end of this time, tadpoles were in developmental stages 31 to 33. After this period, the tadpoles were euthanized with buffered MS-222 (Ethyl 3-aminobenzoate methanesulfonate; Merck, Rahway, New Jersey, U.S.) and the spectral composition of their dorsal pigmentation was measured using a spectrophotometer (Model CM-2600d, Konica Minolta, Marunouchi, Chiyoda, Tokyo, Japan). Tadpoles were gently padded dry with a paper towel, and the measurements were performed by gently pressing the sensor on the skin on the dorsum, just anterior to the base of the tail insertion. Three readings per individual were taken, slightly repositioning the sensor a few millimetres every time. The spectrophotometer was set to exclude specular reflectance, illuminant D65 with UV adjustment 100% and observer angle 10°. We used the same technique to quantify background colour. We took three randomly placed measurements of the reflectance spectra of the inside walls of each container with the same settings. Containers were dried completely and the outside wall relative to where the measurements were taken was pressed up against the lab bench so as not to allow any light transmission through the container wall.

The spectrophotometer measurements of the tadpoles and backgrounds were digitized using the SpectraMagic NX software and exported as a plain text file. The raw reflectance per wavelength readings (ranging from 360 to 740 nm) were then processed and analysed using the R package ‘pavo’ v. 2.3.0^71^. First, raw spectra were trimmed to include only the visible spectrum (400 to 700 nm), and smoothed to remove noise with span = 0.75. The spectra derived from measurements of the tadpoles and backgrounds are provided as Supplementary Figs. 1 and 2. To characterize spectra shapes, we summarized the reflectance spectra using three representative colorimetric parameters to describe hue (H1), chroma or saturation (S8) and brightness (B2) following Montgomerie^72^. H1 is the maximum peak of reflectance in the wavelength, S8 is the difference between the maximum and minimum reflectance divided by the mean reflectance, and B2 is the mean relative reflectance over the entire spectral range.

To confirm that brightness measurements of the dry containers are correlated with the amount of light reaching the bottom of each container when filled with water, we also measured the light intensity in lux using a submersible smartphone (Ulefone Armor 6) and the application Lux Light Meter (Doggo Apps). The phone was inserted, camera first, into the water-filled containers and the measurement was taken approximately 1 cm from the base of the container along a horizontal plain. Lux measurements of the water filled containers were correlated with the brightness measurements (B2) of the spectrophotometer (Supplementary Fig. 3), and we therefore proceeded to use only the spectrophotometer readings to characterize the tadpoles’ environment.

To compare the dorsal pigmentation of the tadpoles with their corresponding backgrounds, we performed a principal components analysis (PCA) in R, using the smoothed spectra binned into 15 nm bins. This PCA captures all aspects of the spectra, including differences in total reflectance (area under the curve). To compare only the shape of the spectra, we performed a second PCA on spectra normalized to have a mean reflectance of 0. This effectively eliminates differences attributed to brightness. We used Euclidean distances between the tadpoles and their corresponding backgrounds in the multidimensional PCA space as a relative measure of background matching accuracy. We also fitted linear regression models in R to test for correlations between the summary colorimetric parameters (H1, S8 and B2) of the tadpoles and their corresponding backgrounds.

### Morphometric analysis

To analyse the potential relationship between changes in pigmentation and morphology of tadpoles, the lateral profile of five black and five white tadpoles was photographed with a digital camera, shortly before euthanasia. Tadpole shape was then quantified using geometric morphometrics. Nine fixed landmarks and thirteen sliding semilandmarks (see Supplementary Fig. 4) were digitized using tpsDig2 v2.31^73^. To remove potential effects of bending (i.e. variation in tadpoles’ curvature unrelated to the experimental treatments), tadpole shape was adjusted by fitting a quadratic curve with the “unbend specimens” module in tpsUtil v1.47^74^. Shape variables were obtained from landmark and semilandmark coordinates applying Generalized Procrustes Analysis^75,76^ using the R package geomorph v4.0.2^77^. This method translates individual coordinates to the origin, scales them to unit-centroid sizes, and rotates them using least-square criterion until the coordinates align as closely as possible. Principal components analysis (PCA) on the Procrustes shape coordinates was performed to visualize the first two axes of shape divergences. We then tested the significance of treatment-associated (dark versus light backgrounds) differences in shape using a Procrustes ANOVA with 1000 permutations and residual randomization.

### Pigmentation reversibility

Six additional tadpoles individualized at Gosner stage 25 were reared in black or white containers (three each) in the same way as previously described for 59 days to elicit the darkest and lightest possible phenotypes. The tadpoles were then reciprocally transplanted to investigate the ability to reverse their pigmentation. As repeatedly manipulating the tadpoles and taking them out of the water for spectrophotometric measurements was deemed too stressful for the animals, we took standardized photos to quantify changes in pigmentation over time. Tadpoles were first photographed laterally, then momentarily placed in a shallow, grey container filled with water to approximately the height of the tadpole (3 cm). Photos were then taken of the dorsum with a Nikon D7500 fitted with a 40 mm 2.8f Nikkor macro lens, mounted on a tripod with an external flash. The exposure (1/160), aperture (f20) and white balance (6300 K) were set to manual and photos were saved as RAW images. A colour checker (www.greywhitebalancecolourcard.co.uk) was included in the photograph to ensure standardized conditions.

Each individual was photographed daily during the first 7 days and then every third or fourth day for a total of 60 days. At this stage, the last tadpole had reached Gosner stage 42 (emergence of forelimbs). Two tadpoles reached this stage 4 days earlier (one originally dark and one originally light tadpole), and measurements were therefore also stopped earlier. Photos were then digitally processed. First, the colour checker was used to calibrate the white balance and exposure in Adobe Lightroom v10.1.1. The lateral photos were then used to generate a timelapse video using the Morph Age software (https://creaceed.com/morphage) for illustrative purposes. The dorsal photos were used for quantifying pigmentation change. Specifically, using Fiji ImageJ v2.1.0^78^, we selected a standardized rectangular area on the tadpoles’ dorsum, defined by the posterior edges of the eyes and the posterior edges of the body (excluding the tail). The red, green and blue reflectance histograms (ranging from 0 to 255) of this area, as well as the means for each colour channel were extracted using the colour histogram function and exported. Using R, the three channels were combined into a single relative luminance metric, by weighing the mean of each channel according to its perceived brightness (luma = 0.2126*red + 0.7152*green + 0.0722*blue). This luminance was then plotted over time. To find the time point when changes in luminance plateaued (i.e. when minimum or maximum pigmentation was reached), we used a quadratic plateau model using the nonlinear least-squares function in R together with a self-start function SSquadp3xs() from the nlraa v1.2 package^79^ to estimate the starting parameters. This is a segmented, nonlinear regression model, which fits a quadratic model up to a join point, after which it fits a zero-slope plateau.

### Oxidative stress

Estimates of oxidative stress require whole-body homogenates and so we were unable to re-use the same tadpoles as for the rest of the experiment. We therefore repeated the experiment with twelve additional tadpoles, six raised in black and six in white containers. We measured the activity of four antioxidant enzymes in tissue of dark vs. light tadpoles: superoxide dismutase (SOD), catalase (CAT), glutathione reductase (GR) and glutathione peroxidase (GPx). We also measured total glutathione, the ratio of reduced glutathione (GSH) to oxidized glutathione (GSSG) as a measure of oxidative stress and malondialdehyde (MDA) concentration as a measure of oxidative damage via a thiobarbituric acid reactive substances assay. Tadpoles were euthanized, eviscerated and snap-frozen in their entirety in liquid nitrogen. Samples were stored at − 80 °C until further processing. Samples were thawed in a buffered Tris–HCl solution (100 mM Tris–HCl with 0.1 mM EDTA, 0.1% triton X-100, pH 7.8 and 0.1 mM PMSF) to inhibit proteolysis, and homogenized. For each sample we estimated total protein content using standard Bradford’s protocol^80^. We then assessed activity of antioxidant enzymes as in Burraco et al.^65^. In brief, we estimated catalase activity indirectly using the method by Cohen and Somerson^81^, based on the reduction of potassium permanganate. We quantified superoxide dismutase activity according to Cord McCord and Fridovich^82^ based on ferrocythocrome C reduction. We also quantified the amount of malondialdehyde derived from lipid peroxidation, as a function of its reaction to thiobarbituric acid following Buege and Aust^83^. Finally, we also determined the proportion of reduced to oxidized glutathione (GSH and GSSG, respectively) following Galván et al.^84^.

Differences in oxidative damage as indicated by levels of thiobarbituric acid and in non-enzymatic antioxidant capacity (GSH to GSSG ratio) between dark and light tadpoles were tested using two-tailed Student’s *T* tests in R. Differences in enzyme levels were explored using a Principal Component Analysis followed by a *T* test on the first principal component.

### Raman spectroscopy

We used Raman spectroscopy to identify the pigments responsible for producing the differences in colour of dark and light tadpoles. Eumelanin is supposedly the main melanin in amphibians, although pheomelanin has also been detected in adults and larvae of at least one amphibian species^54^. The relative importance of eumelanin and pheomelanin to the pigmentation of an organism has relevant physiological consequences, and it is therefore key to characterize the presence or absence of these two pigments. In particular, the synthesis of the sulphurated form of melanin, termed pheomelanin, is limiting in vertebrates during scenarios of environmental oxidative stress due to the incorporation of thiols to the pigment structure and the consequent consumption of a key antioxidant resource such as cysteine^85^. Eumelanin-based pigmentation may also have limiting physiological consequences due to its potential to increase thermal stress, this being particularly relevant to homeotherms^86^.

Dispersive Raman spectroscopy can detect pigment molecules in solid samples at concentrations as low as 0.05–0.1% (w/w)^87,88^. Three dark and three light tadpoles (reared in black and white containers for 40 days respectively) were analysed using a Thermo Fisher DXR confocal dispersive Raman microscope (Thermo Fisher Scientific, Madison, WI, USA) operating at the National Museum of Natural Sciences (MNCN, CSIC) in Madrid, Spain. The system was run with the OMNIC software v8.1, from Thermo Fisher Scientific. The system has a point-and-shoot Raman capability of 1 μm spatial resolution. Tadpoles were euthanized, dabbed dry with paper towels and each, whole animal was placed on a glass microscopy slide. We used an excitation laser source at 780 nm and a slit aperture of 50 μm to analyze the wavenumber range of 300–2500 cm^–1^. To avoid the burning of samples and to optimize results, we used a 50 × confocal objective lens, a laser power of 10 mW, and an integration time of 6 s and 2 accumulations. Three measurements of the dorsal pigmentation per specimen were taken, slightly repositioning the tadpole between each measurement.

## Supplementary Information

Supplementary Video 1.

Supplementary Video 2.

Supplementary Information.

## Data Availability

The underlying data of this study are made available in the supplementary information and in a public repository available at: https://doi.org/10.5281/zenodo.7788628.
