# Supplementary material for: Background matching through fast and reversible melanin-based pigmentation plasticity in tadpoles comes with morphological and antioxidant changes
Source: Sci Rep. 2023 Jul 26;13:12064. doi: 10.1038/s41598-023-39107-4 (PMC10371988; doi:10.1038/s41598-023-39107-4)
Supplement: Supplementary file 3 — Supplementary Information. [file 41598_2023_39107_MOESM3_ESM.pdf]

Supplementary information

Supplementary Table 1: Brand, product name and description and colour reference of the spray paint used for creating backgrounds.

**Brand**	**Product**	**Description**	**Colour**
Novasol Spray S.A.	PINTY PLUS	Matt Black 9005	Black
Novasol Spray S.A.	PINTY PLUS	Dark Grey 8651	Grey 1
Novasol Spray S.A.	PINTY PLUS	Pearl Grey 8652	Grey 2
Montana Colors S.L.	94	Rita Grey RV-118	Grey 3
Montana Colors S.L.	94	Siberia Grey RV-7047	Grey 4
Montana Colors S.L.	94	Stardust Grey RV-198	Grey 5
Novasol Spray S.A.	PINTY PLUS	Matt White 9010	White
Montana Colors S.L.	94	Madrid Red RV-241	Red
Montana Colors S.L.	94	Fluor Green	Green
Montana Colors S.L.	94	Electric Blue RV-30	Blue

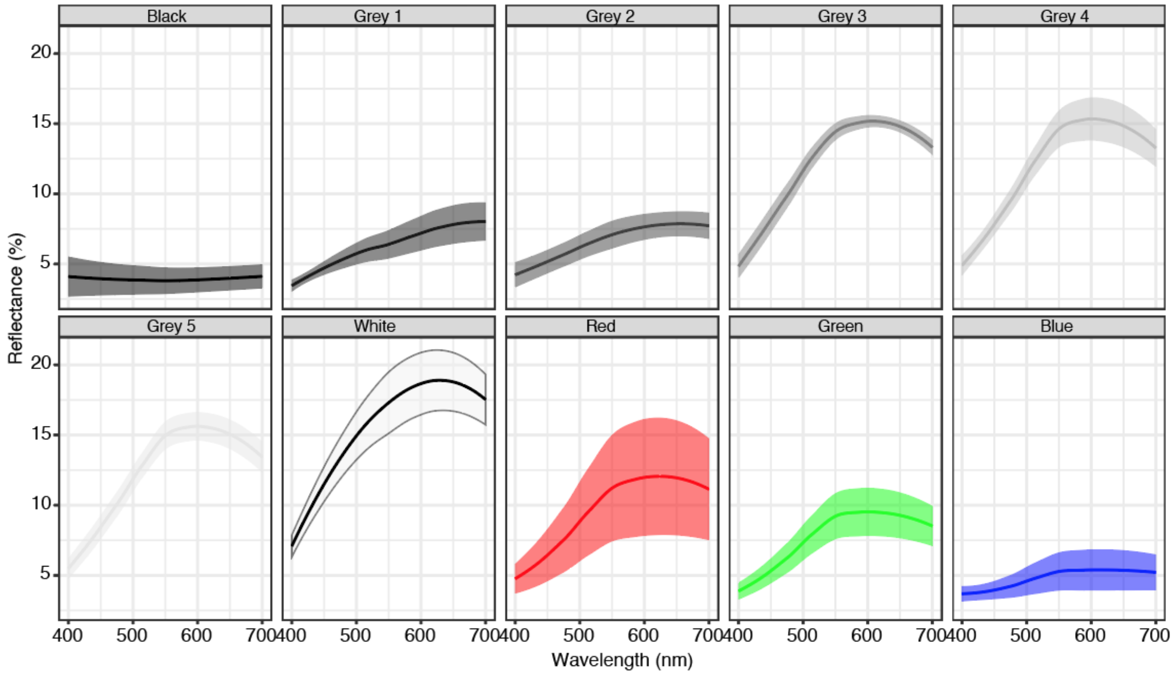

Supplementary Figure 1: Smoothed reflectance curves of dorsal skin of tadpoles reared on different background colours. Panel names indicate background colour and lines and shadings represent mean ± standard deviation of five tadpoles per background colour.

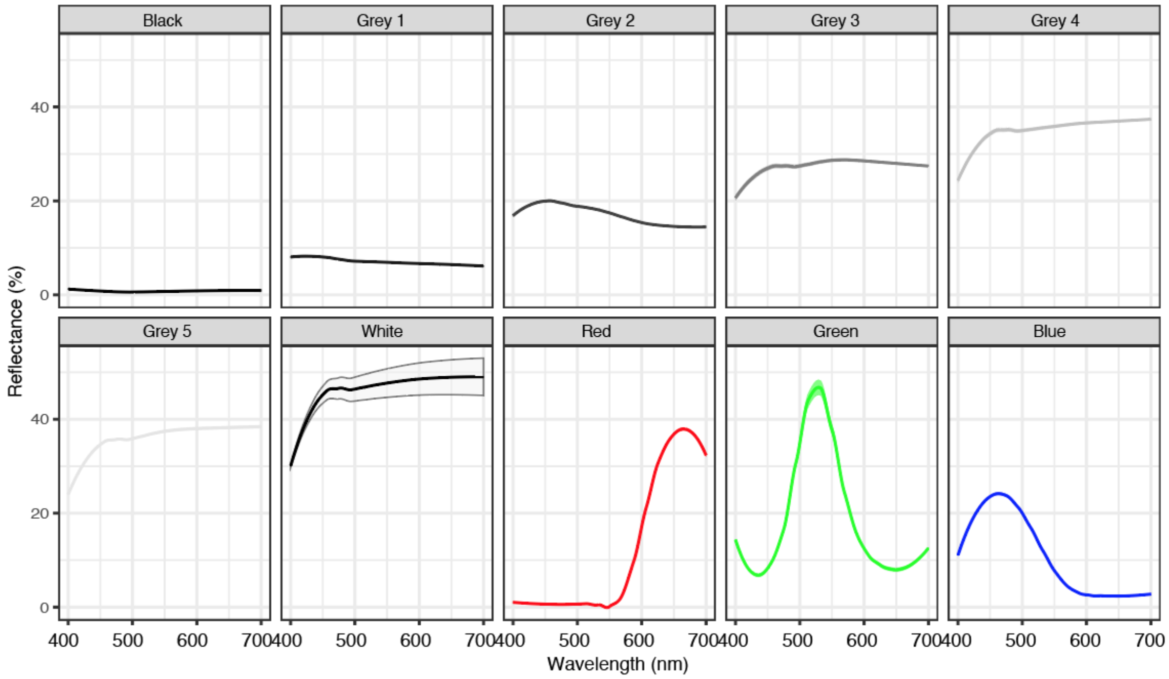

Supplementary Figure 2: Smoothed reflectance curves of different background colours. Panel names indicate background colour and lines and shadings represent mean ± standard deviation of three measurements per background colour.

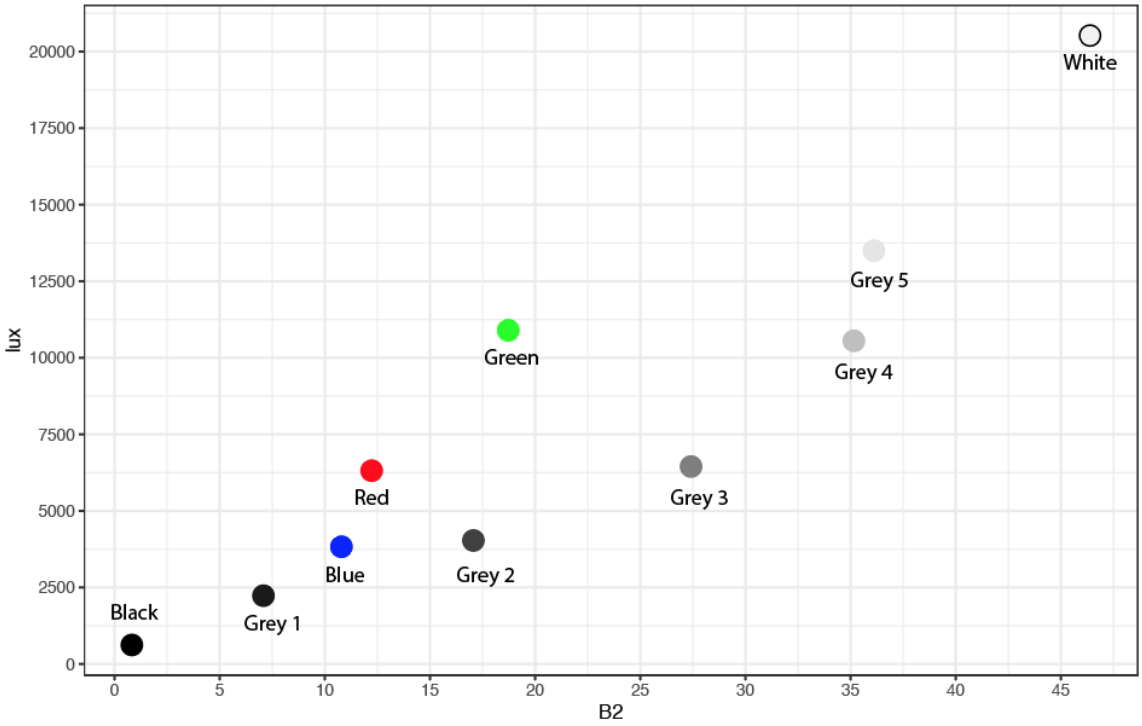

Supplementary Figure 3: Correlation of Lux measurements at the bottom of waterfilled containers with brightness metric (B2) derived from photospectrometer measurements taken on the insides of dry containers. Fill colour of points represents the background colours: black, white, five shades of grey and red, green and blue.

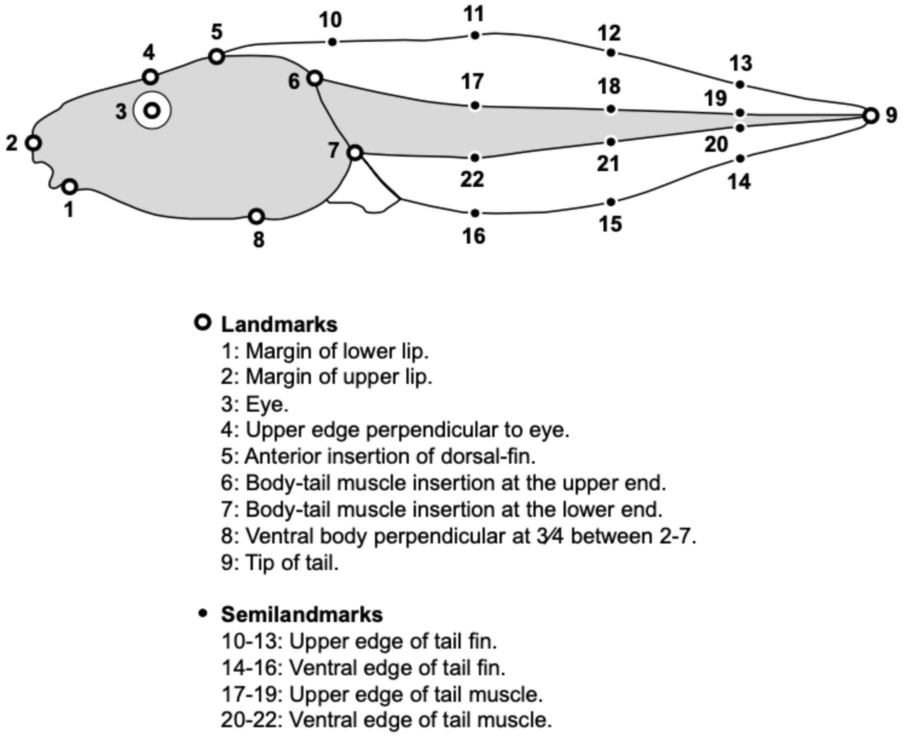

Supplementary Figure 4: Landmarks and semilandmarks used for geometric morphometrics.
